# Characterisation of *bla*_TEM_ genes and types of β-lactamase plasmids in *Neisseria gonorrhoeae* – the prevalent and conserved *bla*_TEM-135_ has not recently evolved and existed in the Toronto plasmid from the origin

**DOI:** 10.1186/1471-2334-14-454

**Published:** 2014-08-22

**Authors:** Ibrahim Muhammad, Daniel Golparian, Jo-Anne R Dillon, Åsa Johansson, Makoto Ohnishi, Sunil Sethi, Shao-chun Chen, Shu-ichi Nakayama, Martin Sundqvist, Manju Bala, Magnus Unemo

**Affiliations:** Department of Laboratory Medicine, WHO Collaborating Centre for Gonorrhoea and other Sexually Transmitted Infections, National Reference Laboratory for Pathogenic Neisseria, Microbiology, Örebro University Hospital, SE-701 85 Örebro, Sweden; Department of Microbiology and Immunology, College of Medicine, University of Saskatchewan, Saskatoon, Saskatchewan Canada; Department of Clinical Microbiology, Central Hospital, Växjö, Sweden; National Institute of Infectious Diseases, Tokyo, Japan; Department of Medical Microbiology, Post Graduate Institute of Medical Education and Research (PGIMER), Chandigarh, India; National Center for STD Control, Chinese CDC, Nanjing, China; Apex Regional STD Teaching, Training and Research Centre, VMMC and Safdarjang Hospital, New Delhi, India

**Keywords:** Gonorrhoea, Antimicrobial resistance, *bla*_TEM-1_, *bla*_TEM-135_, TEM-1, TEM-135, Rio/Toronto plasmid, Extended-spectrum β-lactamase (ESBL)

## Abstract

**Background:**

Antimicrobial resistance (AMR) in *Neisseria gonorrhoeae* is a major concern worldwide. It has been recently feared that the *bla*_TEM-1_ gene is, via *bla*_TEM-135_, evolving into an extended-spectrum β-lactamase (ESBL), which could degrade all cephalosporins including ceftriaxone. The aims of the present study were to characterize the *bla*_TEM_ genes, types of β-lactamase plasmids, the degradation of ampicillin by TEM-135 compared to TEM-1, and to perform molecular epidemiological typing of β-lactamase-producing *N. gonorrhoeae* strains internationally.

**Methods:**

β-lactamase producing *N. gonorrhoeae* isolates (n = 139) cultured from 2000 to 2011 in 15 countries were examined using antibiograms, *bla*_TEM_ gene sequencing, β-lactamase plasmid typing, and *N. gonorrhoeae* multiantigen sequence typing (NG-MAST). Furthermore, the *bla*_TEM_ gene was sequenced in the first described Toronto plasmid (pJD7), one of the first Asian plasmids (pJD4) and African plasmids (pJD5) isolated in Canada. The degradation of ampicillin by TEM-135 compared to TEM-1 was examined using a MALDI-TOF MS hydrolysis assay.

**Results:**

Six different *bla*_TEM_ sequences were identified (among isolates with 125 different NG-MAST STs), i.e. *bla*_TEM-1_ (in 104 isolates), *bla*_TEM-135_ (in 30 isolates), and four novel *bla*_TEM_ sequences (in 5 isolates). The *bla*_TEM-1_ allele was only found in the African and Asian plasmids, while all Rio/Toronto plasmids possessed the *bla*_TEM-135_ allele. Most interesting, the first described gonococcal Toronto plasmid (pJD7), identified in 1984, also possessed the highly conserved *bla*_TEM-135_ allele. The degradation of ampicillin by TEM-135 compared to TEM-1 was indistinguishable in the MALDI-TOF MS hydrolysis assay.

**Conclusions:**

*bla*_TEM-135_, encoding TEM-135, is predominantly and originally associated with the Rio/Toronto plasmid and prevalent among the β-lactamase producing gonococcal strains circulating globally. *bla*_TEM-135_ does not appear, as previously hypothesized, to have recently evolved due to some evolutionary selective pressure, for example, by the extensive use of extended-spectrum cephalosporins worldwide. On the contrary, the present study shows that *bla*_TEM-135_ existed in the Toronto plasmid from its discovery and that *bla*_TEM-135_ is highly conserved (not further evolved in the past >30 years). Nevertheless, international studies for monitoring the presence of different *bla*_TEM_ alleles, the possible evolution of the *bla*_TEM-135_ allele, and the types of β-lactamase producing plasmids, remain imperative.

**Electronic supplementary material:**

The online version of this article (doi:10.1186/1471-2334-14-454) contains supplementary material, which is available to authorized users.

## Background

Gonorrhoea is the most prevalent bacterial sexually transmitted infection (STI) globally, according to the latest estimates by the World Health Organization (WHO) [1]. No vaccine is available and, accordingly, appropriate prevention, and particularly effective diagnosis and antimicrobial treatment are the cornerstones for control of gonorrhoea. *Neisseria gonorrhoeae* has developed resistance to all antimicrobials previously recommended for first-line empiric monotherapy, such as penicillins, tetracyclines, macrolides and fluoroquinolones [2–5]. Clinical resistance to the extended-spectrum cephalosporins (ESCs) has also been verified in the latest years in many countries [6–19]. Furthermore, the first three extensively-drug resistant (XDR [3]) gonococcal strains were recently described; all displayed a high-level of resistance to ceftriaxone, the last remaining option for empiric first-line antimicrobial monotherapy in most countries globally [9, 18, 20]. All the reported decreased susceptibility and resistance to ESCs has been due to an accumulation of chromosomal resistance determinants [2, 4, 5].

In 1976 the first β-lactamase producing *N. gonorrhoeae* strains, resulting in high-level resistance to penicillins but not affecting the MICs of ESCs, were reported [21, 22]. These strains produced the traditional TEM-1 β-lactamase that hydrolyses the cyclic amide bond in the β-lactam ring [23]. β-lactamase producing gonococcal strains are currently widespread internationally [2, 4]. The *bla*_TEM_ genes are located on a family of related β-lactamase plasmids, of which the most frequently described have been the Asian, African, and the mainly indistinguishable Rio and Toronto plasmids (named based on their epidemiological origin) [4, 24–26]. However, other types of β-lactamase producing plasmids have also been described in gonococci, e.g. Nimes, New Zealand, Australian and Johannesburg [4, 26–28]. The Asian plasmid has been considered to be the ancestral plasmid from which the other plasmids evolved through deletions and/or insertions. Accordingly, these β-lactamase producing plasmids may be characterised as either deletion derivates of the Asian plasmid (Africa, Rio/Toronto and Johannesburg) or insertion derivatives of either the Asian (New Zealand) or African (Nimes) plasmids [4, 24–27].

Worryingly, the *bla*_TEM-1_ gene, encoding the TEM-1 β-lactamase, needs only a few specific single nucleotide polymorphisms (SNPs) to evolve into a gene encoding an extended-spectrum β-lactamase (ESBL), which could degrade all ESCs including ceftriaxone [29–32]. The spread of a potent ESBL, which also degrades ceftriaxone, in the gonococcal population might rapidly result in untreatable gonorrhoea in most settings worldwide. It has been hypothesized that the gonococcal *bla*_TEM-1_ has recently evolved into *bla*_TEM-135_, which originally was identified in *Salmonella enterica* subsp. enterica serovar Typhimurium [33] and might be a precursor in the evolution into an ESBL gene [29, 34, 35]. TEM-135 producing gonococcal isolates have been described in 2004 and 2008 in Japan [29], from 2005 to 2007 in Thailand [34, 36], and in 2007 and 2012 in China [35]. TEM-135, which only differs from TEM-1 by one SNP (T→C at position 539) resulting in the amino acid alteration M182T, requires solely one additional specific SNP to evolve into an ESBL such as TEM-20 [29–32, 34]. In those ESBLs, the M182T alteration presumably stabilizes the active site topology reorganized by other mutations, which collaboratively results in the emergence of a stable ESBL [34, 35, 37]. However, there have been few reports, and none outside Asia, regarding surveillance of the different types of *bla*_TEM_ genes and β-lactamase producing plasmids in the gonococcal strains circulating worldwide.

The aims of this study were to characterize the *bla*_TEM_ genes, the types of β-lactamase plasmids, the degradation of ampicillin by TEM-135 compared to TEM-1, and to perform molecular epidemiological typing of β-lactamase-producing *N. gonorrhoeae* isolates cultured in 2000–2011 in 15 countries.

## Methods

### β-lactamase producing *N. gonorrhoeae*isolates

In total, 139 β-lactamase producing *N. gonorrhoeae* isolates were examined, including 136 *N. gonorrhoeae* clinical isolates (collected from 2000 to 2011 in 15 WHO European (n = 40), African (n = 22), American (North and Latin America) (n = 10), Southeast Asian (n = 33) or Western Pacific (n = 31) countries) and three of the 2008 WHO *N. gonorrhoeae* reference strains, i.e. WHO M (isolated in the Philippines, 1992), WHO N (Australia, 2001), and WHO O (Canada, 1991) [38]. All isolates were cultured on selective culture media for 16–18 h at 37°C, in a 5% CO_2_-enriched atmosphere. *N. gonorrhoeae* isolates were identified using characteristic colony morphology, Gram staining, positive oxidase test, a rapid carbohydrate utilization test, and the Phadebact GC Monoclonal Test (Boule Diagnostics AB, Huddinge, Sweden). All examined gonococcal isolates were cultured and stored as part of the routine diagnostics (standard care) and no patient identification information was used. Furthermore, to elucidate when *bla*_TEM-135_ emerged DNA from three previously published strains was also investigated. Those represented the first Toronto plasmid described (pJD7 [39, 40], identified in 1984), one of the first Asian plasmids (pJD4 [39–41], identified in the late-1970s) and one of the first African plasmids (pJD5 [39–41], identified in the late-1970s) isolated in Canada.

### Antimicrobial susceptibility testing

MICs (mg/L) of the isolates for ampicillin, cefixime, ceftriaxone, azithromycin, ciprofloxacin, and spectinomycin were determined using the Etest method (AB bioMérieux, Solna, Sweden), according to the instructions from the manufacturer. Breakpoints for susceptibility, intermediate susceptibility and resistance in accordance to the European Committee on Antimicrobial Susceptibility Testing (EUCAST; http://www.eucast.org) were used. β-lactamase production was identified using nitrocefin solution (Oxoid, Basingstoke, Hants, England). The 2008 *N. gonorrhoeae* WHO reference strains [38] were used for quality control in all antimicrobial susceptibility testing.

### β-lactamase hydrolysis assay

To examine the degradation of ampicillin by TEM-135 compared to TEM-1, a selection of six β-lactamase producing gonococcal isolates (three *bla*_TEM-135_ and three *bla*_TEM-1_ isolates) were investigated using a hydrolysis assay. The non-β-lactamase producing *N. gonorrhoeae* reference strain WHO F [38] was included as negative control. All isolates were cultured on New York City agar media plates and incubated for 16–18 h at 37°C in a 5% CO_2_-enriched atmosphere. The hydrolysis assay was performed as previously described [42] on a Microflex (Bruker Daltonics, GmbH, Germany), by recording spectra in the mass range of 0–1000 Da after incubation for 15 minutes, 1 h, 2 h and 3 h. For calibration, the Peptide calibration standard II (Bruker Daltonics, GmbH, Germany) was used. The peaks utilized for calibration was CCA [M + H] + at 190.05 Da, CCA [2 M + H] + at 379.09 Da and Bradykinin (1–7) peak [M + H] + at 757.40 Da. Generated spectra were manually examined using the Flex Analysis 3.1 software (Bruker Daltonics, GmbH, Germany), and peaks correlating to hydrolyzed or intact ampicillin [42] were identified.

### Plasmid DNA extraction

The QIAGEN Spin Miniprep Kit (QIAGEN, Hilden, Germany) was used to extract and purify plasmid DNA, according to the instructions from the manufacturer. DNA was stored at 4°C prior to subsequent analysis.

### Genomic DNA extraction

Genomic DNA was extracted using the robotized NorDiag Bullet (NorDiag ASA, Oslo, Norway) and the BUGS’n BEADS STI-fast kit (NorDiag ASA, Oslo, Norway), according to the instructions from the manufacturer. DNA was stored at 4°C prior to subsequent analysis.

### Plasmid typing

Multiplex PCR was performed for β-lactamase plasmid typing on all isolates as previously described using the primers BL1, BL2, BL3, and BL4 [25].

### Sequencing of the *bla*_TEM_gene

The entire coding region, including the signal peptide of 23 amino acids (GenBank accession number AAR25033), of *bla*_TEM_ was PCR amplified in a LightCycler real-time PCR System (Roche Molecular Biochemicals, Mannheim, Germany) and subsequently sequenced as previously described [29]. Multiple-sequence alignments of nucleotide and amino acid sequences were performed using the BioEdit (version 5.0.9) software. For comparison and numbering of the amino acid positions, all amino acid sequences identified in the present study were compared to sequences at the Lactamase Engineering Database (http://www.laced.uni-stuttgart.de/) as well as at the β-Lactamase Classification and Amino Acid Sequences for TEM, SHV and OXA Extended-Spectrum and Inhibitor Resistant Enzymes database (http://www.lahey.org/Studies/). The scheme proposed by Ambler et al. [43] was used for numbering of amino acids.

### Molecular epidemiological characterisation using *Neisseria gonorrhoeae*multiantigen sequence typing (NG-MAST)

Amplification and sequencing of the more variable segments of the *porB* and *tbpB* genes examined in NG-MAST [44, 45] was performed as previously described [46]. For assignment of *porB* and *tbpB* allele numbers as well as NG-MAST STs, the NG-MAST website (http://www.ng-mast.net/) was used.

## Results

### Antimicrobial susceptibility of β-lactamase producing *N. gonorrhoeae*isolates (n = 139) from 2000 to 2011

The majority (83.5%, n = 116/139) of isolates were resistant to ampicillin and the remaining 16.5% (n = 23) had an intermediate susceptibility to ampicillin. Furthermore, 80.6% (n = 112) and 6.5% (n = 9) of the isolates were resistant to ciprofloxacin and azithromycin, respectively. Only one isolate (0.7%) was resistant to cefixime (MIC = 0.25 mg/L) and no isolates were resistant to ceftriaxone or spectinomycin.

### β-lactamase plasmid types and *bla*_TEM_alleles

The African-type β-lactamase plasmid was the most common (67.6% [94/139] of isolates), followed by the Rio/Toronto-type plasmid (18.7% [26/139] of isolates) and the Asian-type plasmid in 13.7% (19/139) of isolates (Table 1). No other β-lactamase plasmid types were found.

**Table 1:** **Type of** ***bla***_**TEM**_ **allele**, **β**-**lactamase producing plasmid**, **number of NG**-**MAST STs**, **and year and WHO Region of isolation of** ***N. gonorrhoeae*** **isolates cultured in 2000**–**2011 in 15 countries**

β-lactamase encoding gene (No. of isolates)	β-lactamase producing plasmid types (%)	Number different NG-MAST STs	Year (WHO Region) of isolation
*bla*_TEM-1_ (104)	African (85.6), Asian (14.4)	93	2000-2011 (All Regions^*a*^)
*bla*_TEM-135_ (30)	Rio/Toronto (86.7), Asian (10), African (3.3),	29	2000-2009 (All Regions^*a*^)
*bla*_TEM_-P14S^*b*^ (2)	African (100)	2	2008 (European)
*bla*_TEM_-P14T^*b*^ (1)	Asian (100)	1	2003 (European)
*bla*_TEM_-E110K (1)	African (100)	1	2003 (African)
*bla*_TEM_-G228S (1)	African (100)	1	2009 (Western Pacific)
*bla*_TEM-135_^*c*^	Rio/Toronto		1984 (American)
*bla*_TEM-1_^*c*^	African		Late-1970s (American)
*bla*_TEM-1_^*c*^	Asian		Late-1970s (American)

^*a*^WHO European, African, American (North and Latin America), Southeast Asian and Western Pacific Region.

^*b*^Amino acid alteration in the 23 amino acids long signal peptide.

^*c*^Previously published strains possessing the first Toronto plasmid described (pJD7 [39, 40]), one of the first Asian plasmids (pJD4 [39–41]) and African plasmids (pJD5 [39–41]) isolated in Canada.

**Figure 1:**
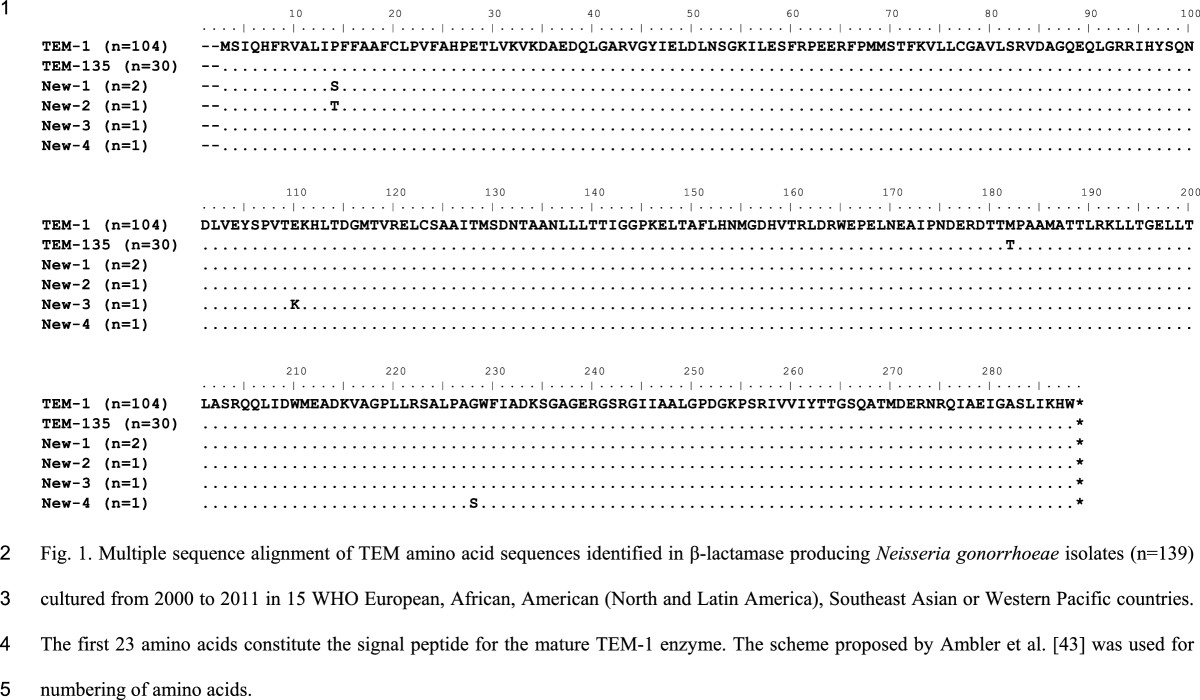
**Multiple sequence alignment of TEM amino acid sequences identified in β-** **lactamase producing** ***Neisseria gonorrhoeae*** **isolates** **(n =** **139)** **cultured from 2000 to 2011 in 15 WHO European,** **African,** **American** **(North and Latin America),** **Southeast Asian or Western Pacific countries.** The amino acids 3-25 constitute the signal peptide for the mature TEM-1 enzyme. The scheme proposed by Ambler et al. [43] was used for numbering of amino acids.

The amino acid sequences of all gonococcal TEM sequences identified in the present study are displayed in Figure 1. Briefly, one-hundred and four isolates (74.8%) possessed the *bla*_TEM-1_ allele, which was identical to the *bla*_TEM-1_ allele in *E. coli* (GenBank accession number AAR25033), and 30 (21.6%) of isolates had an identical *bla*_TEM-135_ allele. Interestingly, all (n = 26) Rio/Toronto plasmids contained the *bla*_TEM-135_ allele. However, the African plasmid and Asian plasmid in one (1.1%) and three (15.8%) isolates also contained the *bla*_TEM-135_ allele. Furthermore, four novel amino acid substitutions in TEM were identified, that is, alterations in the signal peptide (P14T (n = 1) and P14S (n = 2)), and in the TEM coding sequence: E110K (n = 1) and G228S (n = 1), which is close to the substrate binding site. All these new *bla*_TEM_ alleles were possessed by African (n = 4) or Asian (n = 1) plasmids (Table 1).

Most interesting, the first described Toronto plasmid (pJD7 [39, 40]), isolated in 1984, also possessed the *bla*_TEM-135_ allele, while the Asian plasmid (pJD4 [39–41]) and African plasmid (pJD5 [39–41]), isolated in the late-1970s, had the *bla*_TEM-1_ allele.

The mean MIC of ampicillin in TEM-135 and TEM-1 producing isolates was 16 mg/L and 12 mg/L, respectively. Furthermore, the mean MIC of ceftriaxone in the TEM-135 and TEM-1 producing isolates was identical, that is, 0.016 mg/L. The isolates containing the novel *bla*_TEM_ alleles had also low MICs of ceftriaxone (MIC = 0.003-0.006 mg/L).

### Degradation of ampicillin by TEM-135 and TEM-1

In the hydrolysis assay, the MALDI-TOF MS spectra showed that the degradation of ampicillin by TEM-135 and TEM-1 was indistinguishable and both β-lactamases degraded ampicillin in 15 minutes. For the non-β-lactamase producing *N. gonorrhoeae* reference strain WHO F [38], included as negative control, no hydrolysis was observed even after three hours of incubation (Additional file 1: Figure S1).

### Genotyping using *N. gonorrhoeae*multiantigen sequence typing (NG-MAST)

In total, 125 different NG-MAST STs were identified, of which 87 had not been previously described. The most frequent STs were ST6058 (3.5% of isolates), ST6057 (2.9%), and ST1288 (2.2%). The majority of STs (93.6% of the STs) were represented by one isolate, whereas five ST were represented by two isolates. Due to the heterogeneity of the isolates and the many STs identified, it was not possible to correlate any β-lactamase plasmid type to any specific NG-MAST ST. Notably, the 30 gonococcal isolates possessing the *bla*_TEM-135_ allele were assigned to 29 different NG-MAST STs.

## Discussion

The present study investigated the characteristics of *bla*_TEM_ genes and their association with β-lactamase plasmid type, the degradation of ampicillin by TEM-135 compared to TEM-1, and the molecular epidemiology of international β-lactamase-producing *N. gonorrhoeae* isolates. The isolates comprised a large collection of temporally (cultured from 2000 to 2011), geographically (from 15 WHO European, African, American (North and Latin America), Southeast Asian or Western Pacific countries) and genetically diverse (125 NG-MAST STs) gonococcal strains. Clearly, *N. gonorrhoeae* strains producing TEM-1 are widespread and all these strains carried the *bla*_TEM-1_ allele on an African or Asian plasmid. The highly conserved *bla*_TEM-135_ allele was predominantly found on the Rio/Toronto plasmids, i.e. only four of the 30 identified *bla*_TEM-135_ alleles were carried on African (n = 1) or Asian (n = 3) plasmids. In fact, all the identified Rio/Toronto plasmids in 26 genetically highly diverse isolates (26 different NG-MAST STs) contained a *bla*_TEM-135_ allele and according to our best knowledge *bla*_TEM-1_ has only been described in one Rio/Toronto plasmid ever [34]. These results strongly indicate that the Rio/Toronto-type plasmid is the origin of the *bla*_TEM-135_ allele. Interestingly, in the present study the *bla*_TEM-135_ allele was also identified on the first reported Toronto plasmid (pJD7 [39, 40]), found in a gonococcal isolate from 1984. This shows that the *bla*_TEM-135_ allele has not recently evolved and, on the contrary, was present in the Toronto plasmid from its discovery. This might also question the evolutionary origin of the Rio/Toronto plasmids, i.e. that these plasmids emerged directly through a deletion of 2273 bp in the Asian plasmid [25, 27], in which the *bla*_TEM-135_ allele is rare internationally. Nevertheless, as shown in the present study the *bla*_TEM-135_ allele can also be found on African-type and Asian-type plasmids. Thus, it cannot be excluded that those *bla*_TEM-135_ alleles might have evolved from *bla*_TEM-1_ alleles by a SNP in those plasmids. However, considering how conserved the gonococcal *bla*_TEM_ alleles appear to be, a more plausible explanation might be that these *bla*_TEM-135_ alleles were acquired by horizontal transfer from strains possessing the *bla*_TEM-135_ allele on a Rio/Toronto plasmid. This hypothesis is further supported by the lack of antimicrobial selective pressure for the evolution of *bla*_TEM-1_ to *bla*_TEM-135_. Accordingly, similar MICs of ampicillin and ceftriaxone, and of other antimicrobials, were displayed by the TEM-135 and TEM-1 producing isolates, and the two different TEM enzymes showed an indistinguishable degradation of ampicillin in the MALDI-TOF MS hydrolysis assay. Nevertheless, for detailed measurement of the kinetics of the ampicillin hydrolysis appropriate kinetic experiments (Kcat/Km) with a purified protein would be required. All the novel *bla*_TEM_ alleles found in the present study were carried by African (four strains) or Asian (one strain) plasmids, and none of these five isolates had any enhanced MIC of ceftriaxone.

## Conclusions

*bla*_TEM-135_, encoding TEM-135, is predominantly and originally associated with the Rio/Toronto plasmid and prevalent among the β-lactamase producing gonococcal strains circulating globally. *bla*_TEM-135_ does not appear, as previously hypothesized, to have recently evolved due to, for example, some evolutionary selective pressure by the extended-spectrum cephalosporins. On the contrary, *bla*_TEM-135_ existed in the Toronto plasmid from its discovery. The present study indicates that *bla*_TEM-135_ instead is highly conserved (not further evolved in the past >30 years). Accordingly, despite the extensive use of extended-spectrum cephalosporins globally no additional SNP has evolved in *bla*_TEM-135_. The reasons for this remain unknown, however, both the lack of evolutionary positive selection for such SNPs and a decreased biological fitness of gonococcal strains with the resulting TEM alleles might be involved. Even so, only one specific SNP added to the *bla*_TEM-135_ allele would produce an ESBL (e.g. an additional G238S alteration to evolve into TEM-20), which would be able to degrade all extended-spectrum cephalosporins, possibly rendering gonorrhoea an untreatable infection. Accordingly, international studies for monitoring and enhancing our understanding of, in addition to the chromosomal ESC resistance determinants, the presence of different *bla*_TEM_ alleles, the possible evolution of the *bla*_TEM-135_ allele, and the β-lactamase producing plasmids, remain imperative.

## Electronic supplementary material

Additional file 1: Figure S1: β-lactamase hydrolysis assay. MALDI-TOF MS spectra of ampicillin after incubation with *Neisseria gonorrhoeae* (β-lactamase negative, TEM-1 producing and TEM-135 producing) and *Escherichia coli* ATCC 35218 (TEM-1 producing). Ampicillin alone (top) and *N. gonorrhoeae* incubated in water (second from top) are also shown in the figure. Intact ampicillin displayed the peaks of 350.4 Da, 372.4 Da and 394.4 Da while hydrolysed ampicillin displayed the peaks of 324.4 Da, 368.4 Da, 390.4 Da and 412.4 Da. The peak of 324.4 Da was observed with low intensity also in the top-spectrum indicating a slight spontaneous hydrolysis. (DOCX 94 KB)

Below are the links to the authors’ original submitted files for images.

Authors’ original file for figure 1
