# Supplementary material for: Characterisation of blaTEM genes and types of β-lactamase plasmids in Neisseria gonorrhoeae – the prevalent and conserved blaTEM-135 has not recently evolved and existed in the Toronto plasmid from the origin
Source: BMC Infect Dis. 2014 Aug 22;14:454. doi: 10.1186/1471-2334-14-454 (PMC4152594; doi:10.1186/1471-2334-14-454)
Supplement: Supplementary file 1 — Additional file 1: Figure S1: β-lactamase hydrolysis assay. MALDI-TOF MS spectra of ampicillin after incubation with Neisseria gonorrhoeae (β-lactamase negative, TEM-1 producing and TEM-135 producing) and Escherichia coli ATCC 35218 (TEM-1 producing). Ampicillin alone (top) and N. gonorrhoeae incubated in water (second from top) are also shown in the figure. Intact ampicillin displayed the peaks of 350.4 Da, 372.4 Da and 394.4 Da while hydrolysed ampicillin displayed the peaks of 324.4 Da, 368.4 Da, 390.4 Da and 412.4 Da. The peak of 324.4 Da was observed with low intensity also in the top-spectrum indicating a slight spontaneous hydrolysis. (DOCX 94 KB) [file 12879_2014_3753_MOESM1_ESM.docx]

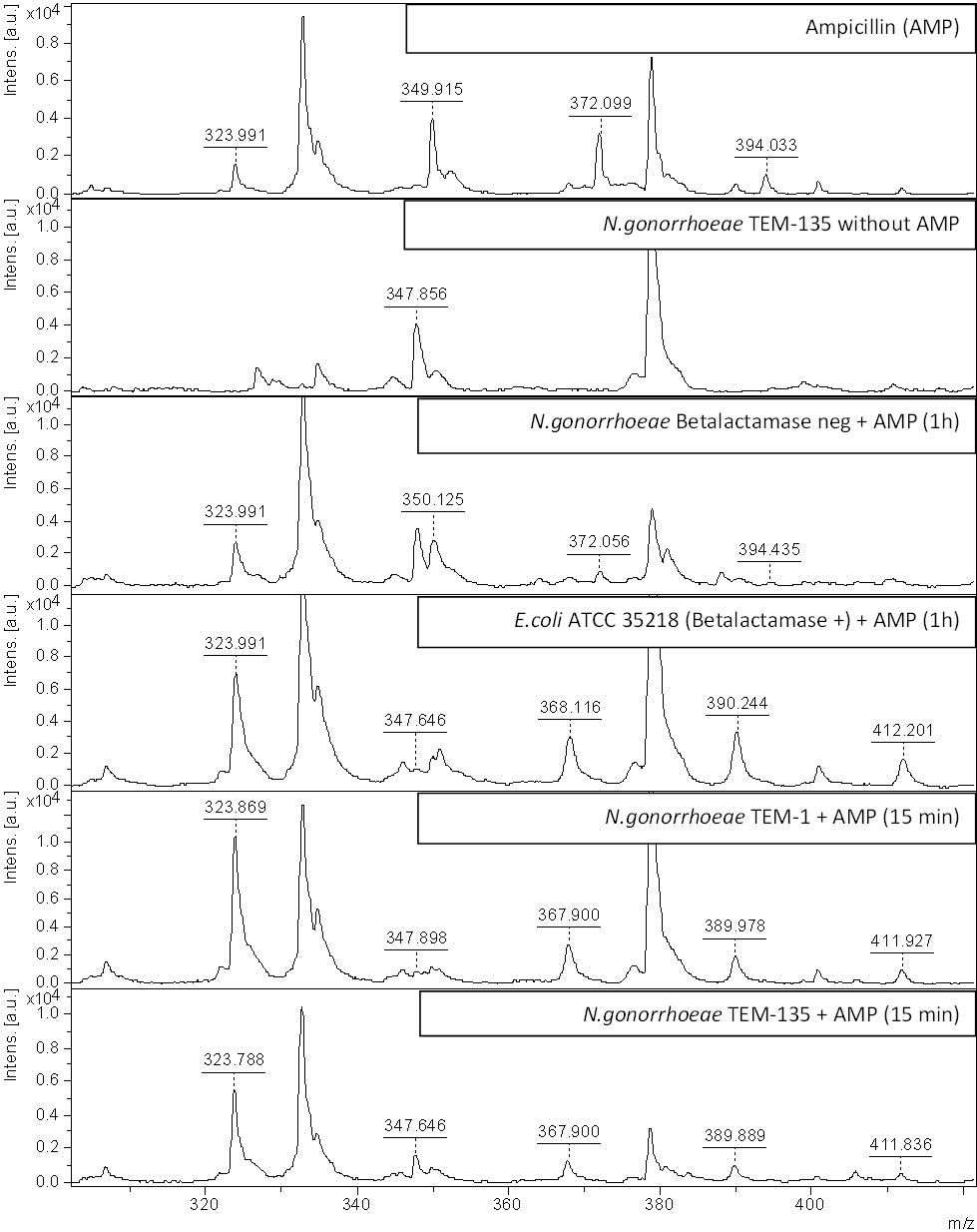


Supplementary Figure 1. **β-lactamase hydrolysis assay.** MALDI-TOF MS spectra of ampicillin after incubation with *Neisseria gonorrhoeae* (β-lactamase negative, TEM-1 producing and TEM-135 producing) and *Escherichia coli* ATCC 35218 (TEM-1 producing). Ampicillin alone (top) and *N. gonorrhoeae* incubated in water (second from top) are also shown in the figure. Intact ampicillin displayed the peaks of 350.4 Da, 372.4 Da and 394.4 Da while hydrolysed ampicillin displayed the peaks of 324.4 Da, 368.4 Da, 390.4 Da and 412.4 Da. The peak of 324.4 Da was observed with low intensity also in the top-spectrum indicating a slight spontaneous hydrolysis.
