# Supplementary material for: Characterisation of blaTEM genes and types of β-lactamase plasmids in Neisseria gonorrhoeae – the prevalent and conserved blaTEM-135 has not recently evolved and existed in the Toronto plasmid from the origin
Source: BMC Infect Dis. 2014 Aug 22;14:454. doi: 10.1186/1471-2334-14-454 (PMC4152594; doi:10.1186/1471-2334-14-454)
Supplement: Supplementary file 2 — Authors’ original file for figure 1 [file 12879_2014_3753_MOESM2_ESM.pdf]

```
                            10        20        30        40        50        60        70        80        90       100
                    ....|....|....|....|....|....|....|....|....|....|....|....|....|....|....|....|....|....|....|....|
TEM-1 (n=104)       --MSIQHFRVALIPFFAAFCLPVFAHPETLVKVKDAEDQLGARVGYIELDLNSGKILESFRPEERFPMMSTFKVLLCGAVLSRVDAGQEQLGRRIHYSQN
TEM-135 (n=30)      --..................................................................................................
New-1 (n=2)         --...........S......................................................................................
New-2 (n=1)         --...........T......................................................................................
New-3 (n=1)         --..................................................................................................
New-4 (n=1)         --..................................................................................................

                           110       120       130       140       150       160       170       180       190       200
                    ....|....|....|....|....|....|....|....|....|....|....|....|....|....|....|....|....|....|....|....|
TEM-1 (n=104)       DLVEYSPVTEKHLTDGMTVRELCSAAITMSDNTAANLLLTTIGGPKELTAFLHNMGDHVTRLDRWEPELNEAIPNDERDTTMPAAMATTLRKLLTGELLT
TEM-135 (n=30)      .................................................................................T..................
New-1 (n=2)         ....................................................................................................
New-2 (n=1)         ....................................................................................................
New-3 (n=1)         .........K..........................................................................................
New-4 (n=1)         ....................................................................................................

                           210       220       230       240       250       260       270       280
                    ....|....|....|....|....|....|....|....|....|....|....|....|....|....|....|....|....|....|....
TEM-1 (n=104)       LASRQQLIDWMEADKVAGPLLRSALPAGWFIADKSGAGERGSRGIIAALGPDGKPSRIVVIYTTGSQATMDERNRQIAEIGASLIKHW*
TEM-135 (n=30)      ........................................................................................*
New-1 (n=2)         ........................................................................................*
New-2 (n=1)         ........................................................................................*
New-3 (n=1)         ........................................................................................*
New-4 (n=1)         ...........................S............................................................*
```

Fig. 1. Multiple sequence alignment of TEM amino acid sequences identified in β-lactamase producing *Neisseria gonorrhoeae* isolates (n=139) cultured from 2000 to 2011 in 15 WHO European, African, American (North and Latin America), Southeast Asian or Western Pacific countries. The first 23 amino acids constitute the signal peptide for the mature TEM-1 enzyme. The scheme proposed by Ambler et al. [43] was used for numbering of amino acids.
